# Inherent Immunogenicity or Lack Thereof of Pluripotent Stem Cells: Implications for Cell Replacement Therapy

**DOI:** 10.3389/fimmu.2017.00993

**Published:** 2017-08-18

**Authors:** Arvind Chhabra

**Affiliations:** ^1^Department of Medicine, University of Connecticut Health Center (UCONN Health), Farmington, CT, United States

**Keywords:** human pluripotent stem cells, induced pluripotent stem cells, dendritic cells, immunogenicity, cell replacement therapy

## Abstract

Donor-specific induced pluripotent stem cells (iPSCs) offer opportunities for personalized cell replacement therapeutic approaches due to their unlimited self-renewal potential and ability to differentiate into different somatic cells. A significant progress has been made toward generating iPSC lines that are free of integrating viral vectors, development of xeno-free culture conditions, and differentiation of pluripotent stem cells (PSCs) into functional somatic cell lineages. Since donor-specific iPSC lines are genetically identical to the individual, they are expected to be immunologically matched and these iPSC lines and their cellular derivatives are not expected to be immunologically rejected. However, studies in mouse models, utilizing rejection of teratomas as a model, have claimed that syngenic iPSC lines, especially the iPSC lines derived with integrating viral vectors, could be inherently immunogenic. This manuscript reviews current understanding of inherent immunogenicity of PSC lines, especially that of the human iPSC lines and their cellular derivatives, and strategies to overcome it.

## Major Advances in Pluripotent Stem Cell (PSC) Research Field

Isolation of human embryonic stem cells (hESCs) from early stage embryos (1) and reprogramming of adult somatic cells into induced pluripotent stem cell (iPSC) lines (2–4) have opened up unique opportunities for developing patient-specific cell replacement therapies (CRT), novel drug discovery platforms, and understanding the mechanism of human cell lineages and organ development. Several different types of somatic cells, including terminally differentiated human primary immune cells, have now been successfully reprogrammed and many non-integrating virus-based iPSC derivation approaches have been developed. Among these include methods utilizing recombinant plasmids, episomes, non-integrating viruses such as adenoviruses and sendai viruses, mRNA, and protein delivery-based reprogramming methods that do not require any carrier vector to deliver the reprogramming factors and the microRNA-based reprogramming that does not require exogenous delivery of the reprogramming factors. Several different somatic cell lineages have been derived from donor-specific iPSC lines and a significant progress has been made toward their phenotypic and functional characterization. Significant progress has also been made toward characterizing the mechanism of development and maintenance of pluripotency, effect of reprogramming on genetic and epigenetic landscape of somatic cells, and characterization of the inherent immunogenicity of iPSC lines and their cellular derivatives. However, much remains to be learnt as these issues are critical for generating iPSC lines that have stable genetic and epigenetic profiles and do not exhibit inherently immunogenicity. We here review current understanding of the inherent immunogenicity of PSC lines and their cellular derivatives, potential mechanisms behind inherent immunogenicity of PSC lines, and strategies to overcome potential immune rejection of PSC lines and their cellular derivatives, with a special emphasis on human hESC and iPSC lines and their cellular derivatives.

## Inherent Immunogenicity of Mouse PSC Based on Teratoma Rejection

Since iPSC lines are the genetic replica of somatic cells they are derived from, donor-specific iPSC lines are expected to be immunologically matched to the donor and the cellular derivatives derived from them are therefore not expected be immune rejected, unlike allogenic tissue and organ transplants. However, failure of syngenic mouse iPSC lines to generate teratomas, a method used to validate the differentiation potential of PSC lines, in immune competent mice was recently used as a readout for their inherent immunogenicity by Zhao et al. (5). Authors first showed that B6 syngenic mESC could form teratomas in immune competent B6 mice without any detectable T cell infiltration, but allogenic 129/sv mESC could not, unlike the SCID mice where both the mESC could form teratomas. Subsequently, authors used syngenic iPSC lines to generate teratomas and found that the iPSC lines derived by genome integrating retrovirus, the V-iPSC (17%), were significantly less efficient in generating teratomas than the non-integrating episomal vector-derived E-iPSC (80%) and the teratomas that did form were infiltrated with T cells, thereby concluding that while both the syngenic iPSC lines were immunogenic, V-iPSCs were more immunogenic than the E-iPSCs (5). By comparing the gene expression profiles of ESC- and E-iPSC-induced teratomas, authors identified nine genes that were over-expressed in iPSC-induced teratomas and found teratoma antigen-specific functional T cells in these animals, suggesting that rejection of iPSC-induced teratomas was mediated by teratoma-associated antigen-directed T cell responses, as the CD4^−/−^ and CD8^−/−^ mice could form teratomas (5).

These findings raised significant concerns about the safety of iPSC (6); however, Araki et al. subsequently reported negligible inherent immunogenicity in mESC, syngenic mouse iPSC lines, and their cellular derivatives (7). Authors used seven iPSC lines and five mESC lines and found no statistical differences in their ability to form teratomas or T cell infiltration in teratomas formed from these iPSC lines in 10 independent experiments (7). Authors used chimeric mice derived from the iPSC and mESC lines and engrafted dermal and bone marrow tissues to examine host immune reaction against the iPSC-derived cellular derivatives in syngenic C57Bl6 and allogenic Balb-c mice and showed that the syngenic skin grafts could sustain for more than 10 weeks without much T cell infiltration but the allogenic grafts could not. Engraftment of GFP+ve iPSC or mESC-derived mice bone marrow in wild-type C57Bl6 animals, with or without prior irradiation, also achieved long-term engraftment of engrafted bone marrow-derived T cells, B cells, and granulocytes alongside host cells without any signs of phenotypic abnormalities or immune reactivity (7). Although authors did find T cell infiltration in engrafted cardiomyocytes and the size of teratomas in wild-type BL-6 animals was slightly smaller than in SCID mice, authors did not find any significant differences in inherent immunogenicity profiles of mouse ESC and iPSC lines (7). In agreement with Araki et al. (7), Guha et al. also reported lack of immunogenicity in syngenic C57BL6 mouse iPSC lines by showing that the subcapsular renal space engrafted syngenic iPSC could form teratomas, but allogenic iPSC could not (8). Using *in vitro* proliferation as a readout for T cell response, authors also did not find any difference in the T cell activation profiles of the animals before or after iPSC and ESC engraftment (8).

Interestingly, Todorova et al. have attributed lack of immune rejection observed in renal space system by Guha et al. (8) to the immature phenotype of APC present in the renal space, as coadministration of APC could result in teratoma rejection (9). Furthermore, utilizing humanized mice Zhao et al. supported their initial findings by demonstrating that human iPSC-derived cellular derivatives exhibit differences in their immunogenicity profiles that correlate with their immunogenic antigen profiles (10). Supporting Zhao et al., de Almeida et al. have also reported rejection of mouse iPSC lines (11). Table 1 lists studies reporting immunogenicity or lack thereof of in ESC and iPSC lines. These findings have highlighted the need to characterize the inherent immunogenicity profile of human iPSC lines and their cellular derivatives to develop safe and effective CRT.

**Table 1:** Studies reporting immunogenicity or lack of it in ESC and iPSC lines.

S. No.	Study	Immunogenicity of syngenic mouse-ESC and iPSC lines	Cells used for teratoma induction
1.	Zhao et al. (5)	Immunogenic	1 or 3 × 10^6^
2.	Araki et al. (7)	Non-immunogenic	3 × 10^6^
3.	Guha et al. (8)	Non-immunogenic	1 × 10^6^
4.	Zhao et al. (10)	Immunogenic	2–4 × 10^6^
5.	de Almeida et al. (11)	Immunogenic	1 × 10^6^

## Is Cell Number a Critical Factor in Facilitating Development of PSC-Induced Teratoma?

Interestingly, there are significant differences in the number of transplanted cells used for teratoma induction in abovementioned studies claiming inherent immunogenicity of mouse iPSC lines or lack thereof. While Zhao et al. in their first study used “one or three million” PSC for teratoma induction, without specifying which lines were used at 1 million and which ones at 3 million (5), in their subsequent study in humanized mice authors used 2–4 × 10^6^ cells (10). Araki et al. used 3 × 10^6^ cells (7) and Guha et al. used 1 × 10^6^ in their renal space teratoma induction experiments (8). In this context, it should be pointed out that Koch et al. have shown that the number of PSC used for teratoma induction is an important factor in determining generation of autologous versus allogenic teratomas, such that 1 × 10^6^ allogenic mESC always fail to induce teratoma, 5 × 10^6^ allogenic mESC can form teratomas in some animals (30%), but 20 × 10^6^ allogenic mESC can form teratomas in all the transplanted animals (12). In their humanized mice study, Zhao et al. also reported that only one in six animals injected with 2 × 10^6^ hESC could form teratomas, while all the animals injected with 4 × 10^6^ hESC were able to form teratomas (10). Using 1 × 10^6^ mouse iPSCs, de Almeida also reported failure of these cells to form teratoma (11). Therefore, it is possible that the variability in teratoma formation in these studies is in part due to differences in number of PSC transplanted and an optimum PSC dosage for teratoma induction experiments must be identified to avoid experimental variations.

## Immunosuppressive Properties of Mouse ESC and iPSC Lines

Mouse ESC have been shown to exhibit immunosuppressive properties (12) that has also been confirmed in hESC and human iPSC lines (13, 14). The mESC express mRNAs for MHC molecules but not the corresponding proteins (15), and they can inhibit T cell proliferation and prevent LPS-mediated induction of co-stimulatory molecules in dendritic cells (DCs) in part through a TGF-β-mediated process (12). However, T cells cultured in the presence of mESC can be stimulated following purification suggesting that the mESC-mediated immune suppression is reversible and it does not make T cells anergic. Natural killer (NK) cells target MHC negative cells, but the published data on NK cell-mediated killing of mESC are not clear, as Koch et al. showed that mESC are not susceptible to NK cell-mediated killing (12) while Dressel et al. found that IL-2 activated syngenic, allogenic, and xenogenic NK cells could efficiently kill mESC (16). As mentioned before, systematic characterization of the inherent immunogenicity profile of human iPSC lines, especially the iPSC lines derived from different somatic cell sources and with different iPSC derivation methods, is essential for developing safe and effective CRT, since mouse iPSC lines derived from different somatic cell sources have been shown to harbor somatic cell memory and exhibit differences in their differentiation profiles (17, 18).

This is also important in light of the fact that despite the reprogramming factors discovered in mouse (2) are also sufficient to reprogram human somatic cells (3), significant differences have been found in the downstream genes targeted by these reprogramming factors in mice and humans (19). Ginis et al. reported species-specific differences in cell cycle regulation, apoptosis regulation, and cytokine expression profiles of human and mouse ESC lines (20), and Suh et al. identified 36 microRNAs in hESC that were downregulated in embryoid bodies (EB), some of which were shared with mouse ESC while others were specific for the hESC (21). Differences in mouse and human physiology are also well known now (22). For example, human blood is neutrophil rich (50–70% neutrophils, 30–50% lymphocytes) while mouse blood is lymphocyte rich (75–90% lymphocytes, 10–25% neutrophils) (22). Mouse HSC are c-kit^high^, flt-3^−^ while human HSC are c-kit^low^, flt-3^+^ (23). Humans express 10 toll-like receptors (TLRs) and 22 NOD-like receptors (NLRs), while mice express 12 TLRs and 34 NLRs. Thy1 is a marker for mouse but not human T cells (24), interleukin-7 receptor (IL-7R) deficiency results in loss of both T and B cells in mouse (25) but only of T cells in humans (26) and Zap-70 deficiency results in loss of both CD4^+^ and CD8^+^ T cells in mouse but only of CD8^+^ T cells in humans (27). Caspases also exhibit notable differences, as mouse do not express caspase-10 while humans do and deletion of caspase-8 causes embryonic lethality in mouse but immune deficiency in humans (28, 29). Furthermore, promising treatment modalities in preclinical models do not always produce matching outcomes in humans (30, 31). These differences emphasize that the species variability must be taken into consideration before extrapolating findings in animal models to humans. We now review current understanding of the inherent immunogenicity of hESC and human iPSC lines.

## Immunogenicity of Human hESC and iPSC Lines

The issue of inherent immunogenicity of human pluripotent stem cell (hPSC) lines was first examined with hESC lines, and they were shown to express low levels of MHC class I molecules but no MHC class II molecules (32). Drukker et al. transplanted 1 × 10^6^ hESC in different strains of immune competent mouse and showed that xeno-rejection of hESC is T cell-mediated process (33). To test whether human immune system would reject undifferentiated hESC *in vivo*, authors transplanted human adult skin grafts, hESC and hESC-derived teratoma tissues under the kidney capsule of trimera mouse reconstituted with human PBMC (34) and showed that transplanted hESC and hESC teratoma tissues could form teratomas while transplanted tumor cells were rejected, concluding that the hESC did not trigger allogenic response from human immune system due to immune privileged properties (33). However, when pulsed with antigenic peptides, hESC were killed by antigen-specific CD8^+^ cytolytic T lymphocytes (CTL) (33).

Similar to the mESC, hESC lines have also been shown to exhibit immune privileged properties as they do not induce T cell proliferation in allogenic MLR (13), suppress differentiation and function of human DCs (35). Li et al. examined immune response against transplanted hESC in immune-compromised Prk^−/−^ mice and immune-competent CD1 mice and did not find any sign of immune-reactivity at the transplantation site, evident by lack of granulocytes infiltration in Prk^−/−^ mice and abrogation of granulocyte and lymphocyte infiltration in immune competent mice upon endotoxin administration along with hESC, suggesting that the hESCs exhibit immunosuppressive properties *in vivo* (13). The hESCs failed to trigger T cell response in allogenic MLR assay and treatment with IFN-γ to induce MHC I expression did not facilitate T cell activation by these cells, even upon fixation, suggesting that the hESC possess inherent immune-privileged properties (13). The immunosuppressive effect of hESC has been shown not to be contact dependent as hESC extracts could suppress differentiation and function of human DCs and it was not mediated by IL-10 or TGB-β production (35). Production of arginase-I in tumor microenvironment in known to inhibit T cells by depleting l-arginine from the microenvironment (36) and the hESC-mediated immune suppression has also been shown to utilize this mechanism, as provision of l-arginine mitigates hESC-mediated T cell suppression (37). Utilizing humanized mice, Zhao et al. found that human fetal liver-derived iPSC lines engrafted in animals received some infiltration of reconstituted human immune cells; however, immune response against autologous hiPSC teratomas was much weaker than the allogenic hESC-derived teratomas (10). In addition, expression of CTLA-4-immunoglobulin (CTLA-4-Ig) and PD-L1 in hESC has also been recently shown to prevent their rejection in humanized mice, highlighting the involvement of immune mechanisms in rejection of hESC-induced teratomas (38).

**Figure 1:**
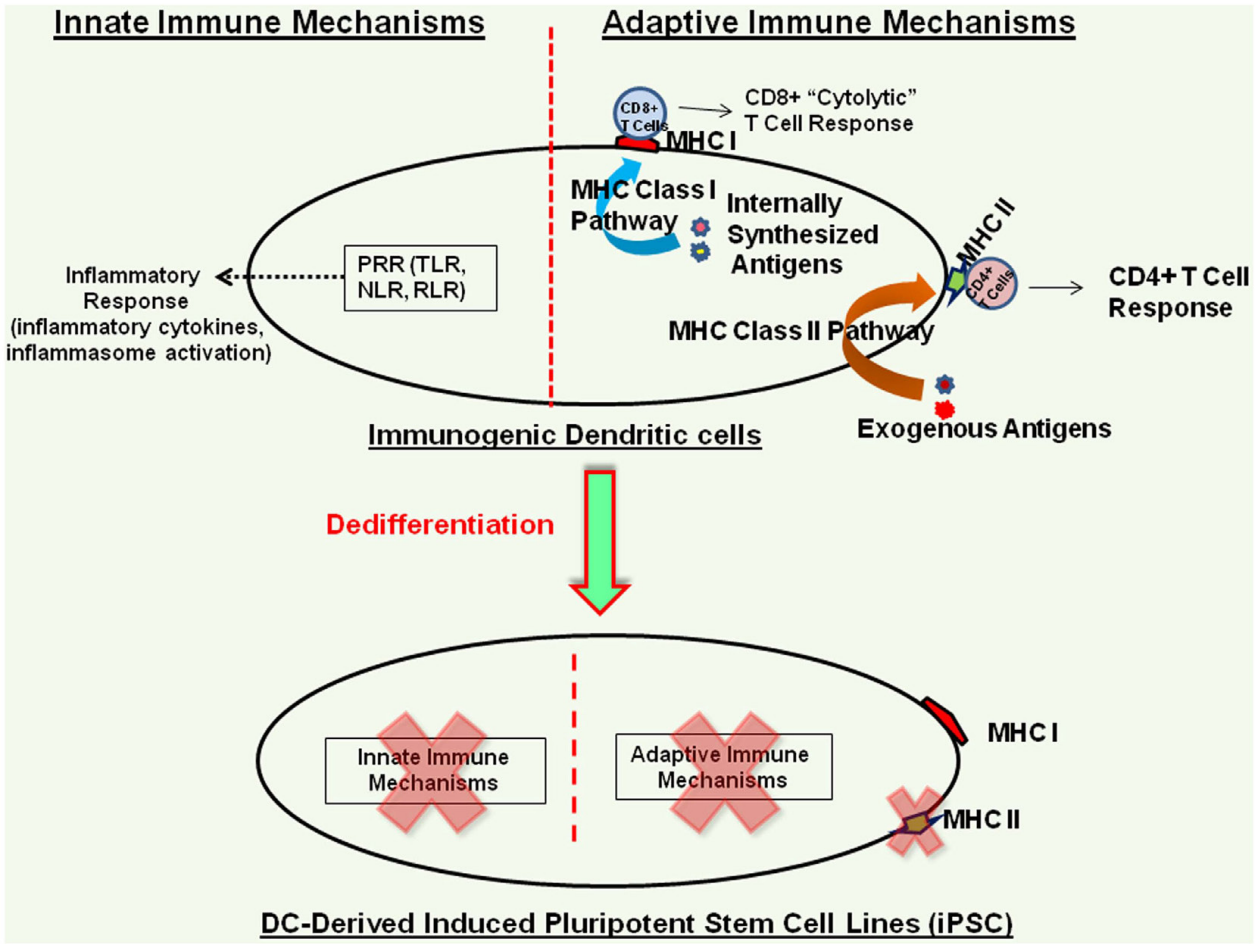
Immunogenicity profile of human terminally differentiated dendritic cell (DC)-derived induced pluripotent stem cell (iPSC) lines. Dedifferentiation of human DCs effectively shuts down their innate and adaptive immune mechanisms; however, these iPSC lines do express low levels of MHC class I molecules (14).

As mentioned before, mouse iPSC lines derived from different somatic cell sources have been found to harbor somatic cell memory and exhibit differential differentiation profiles (17, 18) and despite the usefulness of animal models, significant differences exist between human and mouse physiology (22). Therefore, detailed characterization of the biology and the differentiation potential of human iPSC lines derived from different somatic cell sources is essential to identify the best somatic cell source and the best iPSC derivation method for generating human iPSC lines that exhibit little or no inherent immunogenicity. In this context, iPSC lines derived from human DCs represent an efficient model to characterize the inherent immunogenicity profile of human iPSC lines and their cellular derivatives (14), as DCs harbor well-characterized innate and adaptive immune mechanisms and they serve as the bridge between the innate and adaptive arms of the immune system (39–41). We have recently shown that human DC-derived iPSC lines do not express functional TLR, co-stimulatory molecules, or the antigen presentation machinery, and they fail to trigger TLR-mediated inflammatory cytokine response, inflammasome activation, and T cell activation in MLR assay (14). While DC-derived iPSC lines do express mRNAs of the innate and adaptive response intermediaries, these mRNAs are not translated into functional proteins, highlighting the critical role of DC lineage-specific transcription factors in this process (14). Furthermore, these iPSC lines do not express MHC class II molecules but do express low levels of MHC class I molecules (14), in agreement with findings in hESC lines (13, 33, 42). Utilizing an iPSC line derived from human fibroblast, Lu et al. have also shown that it does not express MHC class II molecules or the co-stimulatory molecules and does not induce T cell proliferation in allogenic MLR (43). Interestingly, despite expressing minimal levels of MHC class I molecules, human DC-derived iPSC lines can efficiently present antigenic peptides to T cells, in agreement with findings in hESC (33). Figure 1 schematically shows the effect of reprogramming on innate and adaptive immune pathways of human peripheral blood-derived DCs (14).

## Immunogenicity of PSC-Derived Cellular Derivatives

In their study showing minimal inherent immunogenicity of syngenic mouse iPSC lines, Araki et al. engrafted skin tissues as well as bone marrow from syngenic iPSC-derived animals and showed that the grafted cells and tissues were accepted by the host, and the bone marrow graft-derived T cells and B cells were also able to coexist with the host immune cells for over 5 months without any sign of immune cross-reactivity (7); however, authors did find immune infiltration at the site of cardiomyocyte injections (7), emphasizing the need to characterize the inherent immunogenicity of different somatic cell lineages and tissues derived from the iPSC lines. Guha et al. engrafted syngenic mouse iPSC, iPSC-derived EB, and cellular derivatives representing three germ layers, the neuronal cells (ectoderm), hepatocytes (endoderm), and endothelial cells (mesoderm), in the subcapsular renal space and did not find any sign of T cell reactivity against the grafts by immunohistochemistry and MLR (8). Morizane et al. transplantated autologous and allogenic iPSC-derived neural cells in non-human primates and found accumulation of CD45^+^ cells at the graft sites 3 months post injection in allograft recipient but not in the autologous graft recipients; however, both the autologous and allogenic grafted neurons survived in the recipients and no detectable IFN-γ or TNF-α were found in the cerebrospinal fluid (CSF) ruling out an active immune response against the grafts (44). Utilizing mouse iPSC-derived endothelial cells (iEC) and comparable somatic cells, the aorta derived endothelial cells (AEC), de Almeida showed that undifferentiated iPSCs were rejected but the differentiated iEC survived and based on the similarities in single-cell transcriptomy profiles of the iEC and AEC, authors concluded that the iPSC-derived cellular derivatives develop immune tolerance properties comparable to self-tolerance mechanisms (11).

With regard to human PSC lines, hPSC-derived EB have been shown to inhibit T cell proliferation, similar to the hESC line (13). Utilizing EB derived from human DC-derived iPSC lines, we have also found that these EB do not express functional proteins for the TLRs, co-stimulatory molecules, or the antigen presentation pathway intermediaries (manuscript under preparation), in agreement with findings in hESC-derived EB (33). Utilizing humanized mice, Zhao et al. found that the smooth muscle cells are inherently more immunogenic than the retinal pigment epithelium, and it correlates with their immune-antigen profiles (10). Taken together, while most of these studies have reported minimal or no immunogenicity of autologous iPSC-derived cellular derivatives in transplanted animals, differences in the immunogenicity profiles of different somatic cells highlight the need to develop efficient models to systematically address this issue. Toward this, detailed characterization of human DC-derived iPSC lines, EB generated from these iPSC lines and more importantly functional APC derived from them could provide useful insights not only toward the inherent immunogenicity profile of human iPSC lines and their cellular derivatives, but also toward development of innate and adaptive immune mechanisms in human DC lineage.

## Potential Mechanisms for Rejection of PSC-Induced Teratoma

**Figure 2:**
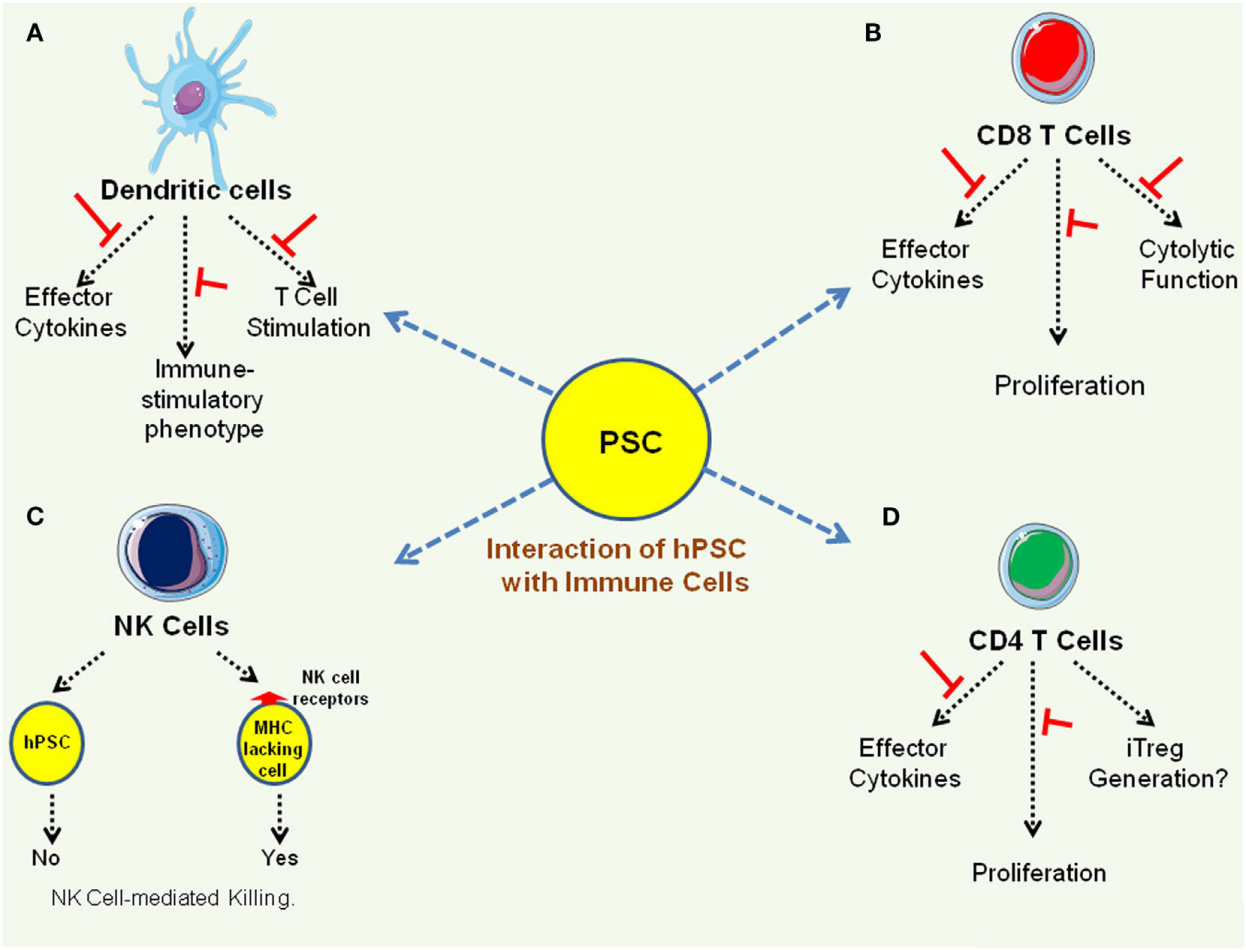
Interaction of immune effectors with human pluripotent stem cell (hPSC) lines. **(A)** Pluripotent stem cell (PSC) suppresses immune stimulatory properties of dendritic cells (35, 37). **(B,C)** PSC suppresses effector function of CD8 T cells as well as CD4 T cells (13, 14). **(D)**. While PSC express very low levels of MHC class I molecules and mouse PSC have been shown to be susceptible to natural killer (NK) cell-mediated killing (16), human PSC are not killed by NK cells as they do not express NK cell receptors (42).

Among the key immune effectors facilitating the development of protective host immune response includes DCs that orchestrate innate and adaptive immune responses and also control self-reactive immune responses, T cells that provide long-lasting cellular immunity, B cells that modulate humoral immunity, and NK cells that target cells lacking MHC molecules. As discussed before, hESC negatively modulate human DC differentiation and function in a contact-independent manner (35). With regard to interaction with T cells, hESC and human iPSC lines do not trigger T cell activation in MLR and suppress T cell function by downregulating the production of effector cytokines and cytolytic activity (13, 14). However, as mentioned before, despite expressing minimal amount of MHC class I molecules (13, 14, 42), hESC and human iPSC lines can efficiently present antigenic peptides to T cells (14, 33, 43). While IL-2-activated mouse NK cells have been shown to kill mESC (16), human hESC are not killed by the NK cells as they do not express NK cell ligands (42). Figure 2 summarizes interaction of hPSC lines with different immune effectors.

**Figure 3:**
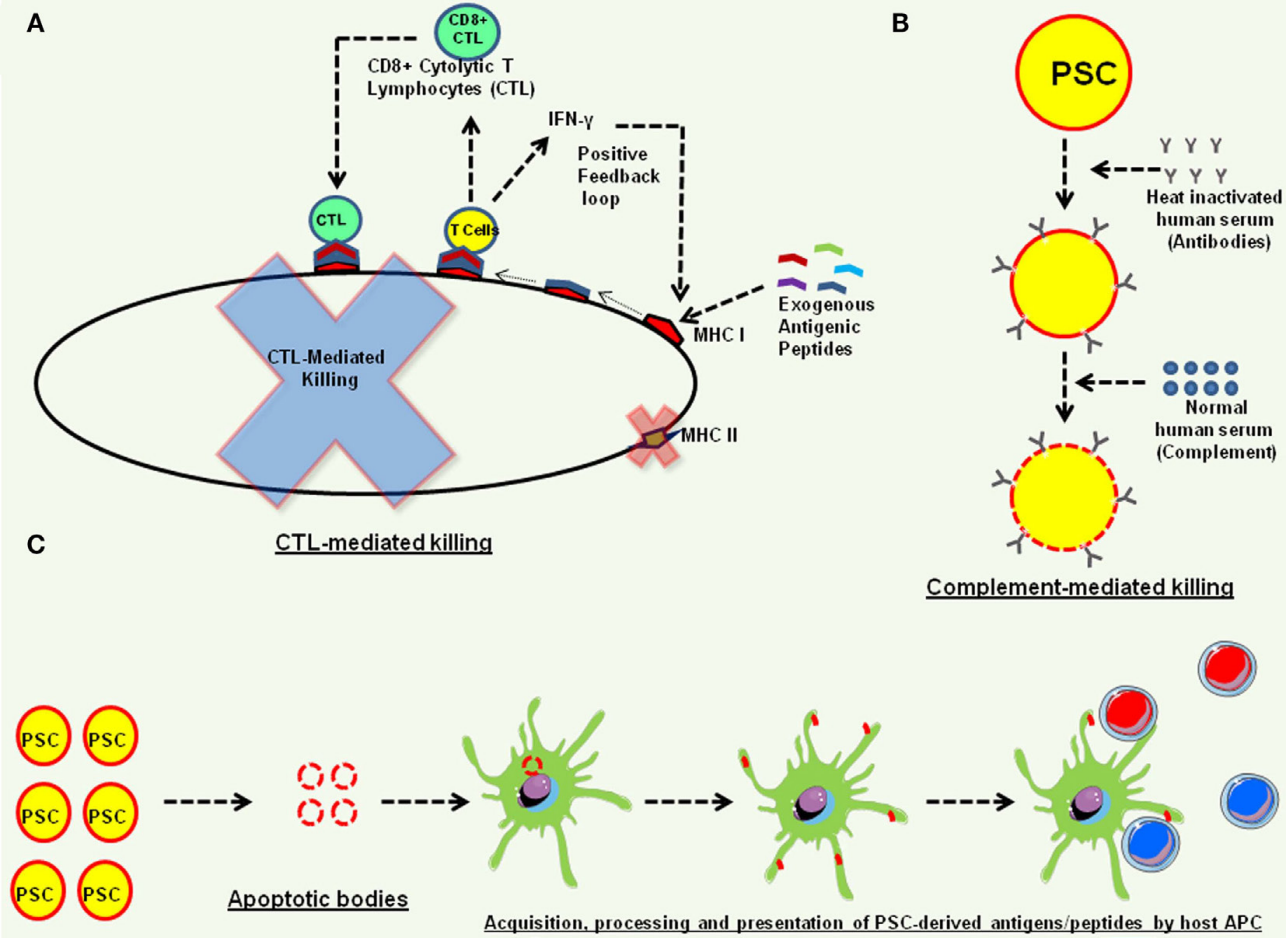
Potential mechanisms of teratoma rejection in animal models. **(A)** The human embryonic stem cell and human induced pluripotent stem cell (iPSC) express low levels of MHC class I molecules (13, 33, 42) that can efficiently acquire antigenic peptides from their microenvironment and present them to CD8^+^ cytolytic T lymphocytes (CTL) that produce effector cytokines, such as interferon-γ (IFN-γ), and also exhibit cytolytic function and kill these pluripotent stem cell (PSC) (14, 43). The IFN-γ can also further induce the expression of MHC class I molecules on human PSC, forming a positive feedback loop (14). **(B)** Complement-mediated killing of PSC. Human PSC are susceptible to alternative complement pathway-mediated killing (45). **(C)** Indirect allo-recognition. Transplanted PSC undergoing apoptosis can be phagocytosed by host antigen-presenting cells, and PSC acquired proteins can be processed and presented to host T cells for alloresponse.

Of note, in normal physiology, PSC do not directly interact with immune effectors and the host immunosuppressive mechanisms prevent immune rejection of developing embryo that harbors 50% foreign DNA. It is possible that the presence of immune infiltrating cells in PSC induced teratomas is due to natural immune surveillance phenomenon and these immune effectors would “ignore” the transplanted PSC and their cellular derivatives due to lack of “non-self antigens” or the “danger signals” or due to immunosuppressive properties of the PSC, as grafted bone marrow-derived T cells and B cells coexist with host immune cells without any sign of immune reactivity (7) and no inflammatory cytokines were detectable in the CSF of neuronal transplants (44). While these findings are encouraging, we now discuss some of the potential mechanisms that might be contributing to the rejection of transplanted ESCs and iPSCs in mouse models (Figure 3). Among these include presentation of antigenic peptides acquired by the transplanted hPSC from their microenvironment to cytolytic T cells that can kill these hPSC in antigen-specific manner (14, 43). Mouse PSC have also been shown to be susceptible to alternative complement pathway-mediated killing that affects their ability to form teratomas, in an inverse correlation to the number of PSC transplanted as complement could block teratoma formation by 1 × 10^5^ but not with 1 × 10^6^ PSC, and the C3^−/−^ mice could form teratomas much faster than the C3^+/+^ mice in part due to deficiency of sialic acid in the PSC (45). In addition, while hESC and iPSC lines do not express MHC II molecules, it is possible that the MHC class II molecules in hPSC are improperly processed and are eventually degraded, thereby generating a repertoire of peptide epitopes that along with other hPSC-derived proteins are acquired by the host APC from apoptotic hPSC through well-known mechanisms (46) and are presented to T cells facilitating an allogenic response, a phenomenon known as indirect allogenic recognition (47).

## Strategies to Overcome Inherent Immunogenicity of PSC

Immunosuppressive drugs (48) as well as immune modulators inhibiting T cell activation (49) have been shown to facilitate acceptance of hESC xeno-transplants in mouse models. Swijnenburg et al. utilized clinically available immunosuppressive drugs, calcinurin inhibitor [tacrolimus (TAC)], target of rapamycin inhibitor [sirolimus (SIR)], and antiproliferative agent (mycophenolate mofetil) and showed that the combination of SIR and TAC could facilitate long-term survival of transplanted hESC by immunosuppressive effect on both the cellular as well as humoral arms of the host immune system (48). However, despite improving the survival of transplanted hESC, it could not facilitate their engraftment beyond 28 days (48). Pearl et al. showed that the combination of CTLA-4-Ig fusion protein, anti-CD40 ligand (anti-CD40L) and anti-lymphocyte function-associated antigen-1 (anti-LFA-1) antibody-mediated short-term immune suppression, to block T cell activation molecules and to provide inhibitory signals, could facilitate engraftment of allogenic/xenogenic hESC as well as iPSC beyond 28 days, through immunosuppressive effect on CD4^+^ and CD8^+^ effector T cells and induction of regulatory T cells (49). As mentioned before, expression of CTL-A4-Ig and PD-L1 in hESC has also been shown to protect them from immune rejection in humanized mice (38); however, it should be emphasized here that constitutive expression of these molecules might not be a good strategy for engrafting iPSC-derived immune cells, as it could compromise their effector function profiles. Tolerance to allografts has also been achieved in animal models through mixed hematopoietic chimerism, whereby donor HSC are engrafted in the host under immunosuppressive conditions and the cells derived from these HSC orchestrate tolerance to allografts (50). In addition, incorporation of regulatory T cells is also known to facilitate allograft acceptance (51).

**Figure 4:**
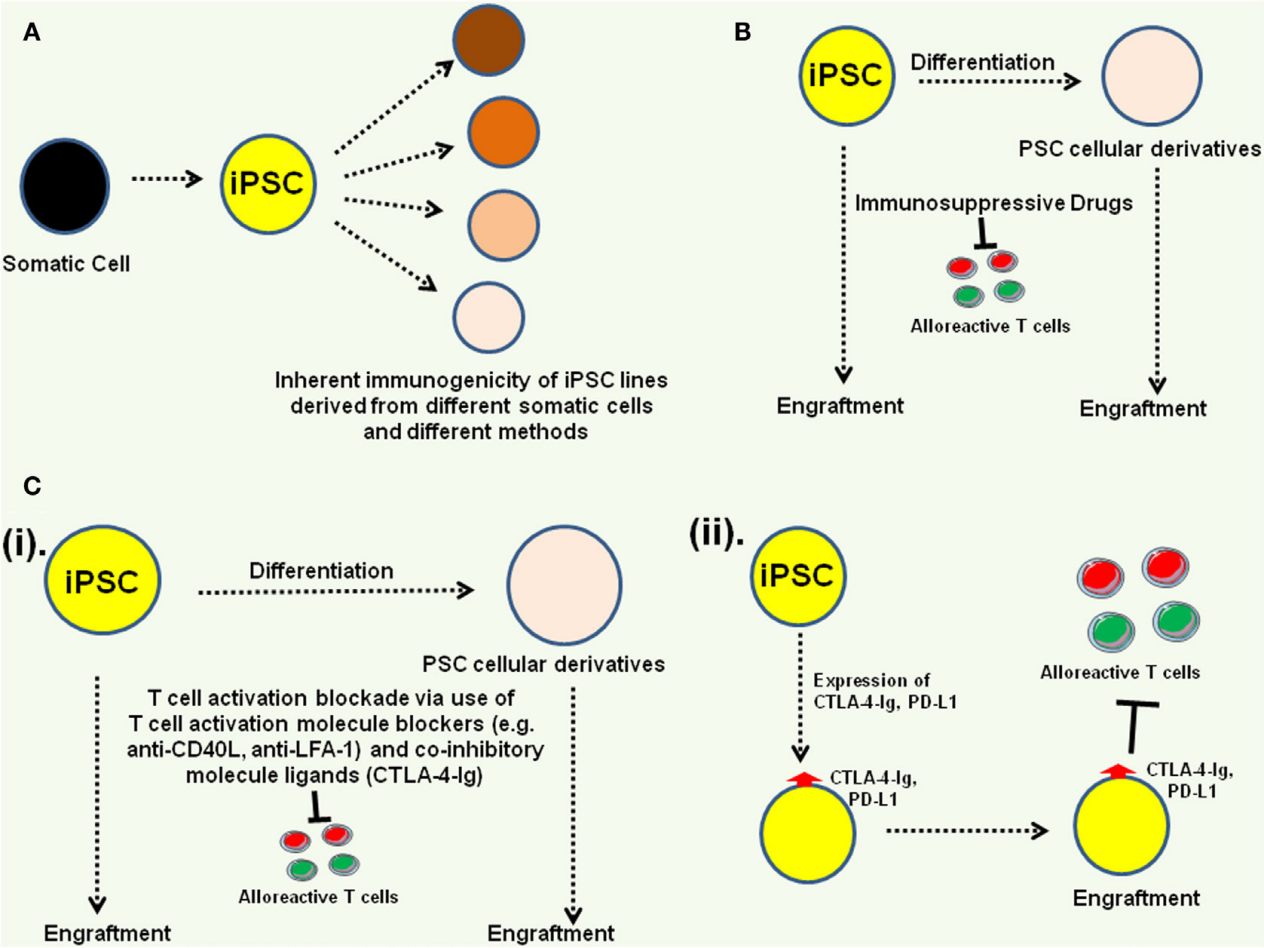
Approaches to overcome potential immunogenicity of induced pluripotent stem cell (iPSC) and their derivatives. **(A)** Identification of best iPSC derivation methods and best somatic cells for generating iPSC lines that exhibit no or very low inherent immunogenicity profiles. **(B)** Incorporation of immunosuppressive drugs can facilitate pluripotent stem cell (PSC) engraftment (48). **(C)** Blocking immune cell activation can also facilitate PSC rejection (49). Among the approaches, T cell activating molecule blockers [e.g., anti-CD40 ligand (anti-CD40L), anti-lymphocyte function-associated antigen-1 (anti-LFA-1)], and co-inhibitory molecule activators [CTLA-4-immunoglobulin (CTLA-4-Ig)] could be administered as adjuvants (i) or co-inhibitory molecule ligands (e.g., CTLA-4-Ig, PD-L1) could be expressed on the PSC (ii) to engage co-inhibitory molecules on host T cells (38).

In summary, while donor-specific human iPSC lines have been shown to exhibit immune suppressive properties and several ESC and iPSC-derived cellular derivatives have been successfully transplanted in animal models without immunological complications, it is possible to overcome host immune rejection mechanisms to facilitate engraftment of iPSC-derived cells/tissues (Figure 4). However, it would be best to optimize the iPSC-derivation methodologies and identify best somatic cells to generate donor-specific iPSC lines that exhibit little or no inherent immunogenicity. Detailed characterization of the inherent immunogenicity profiles of iPSC-derived somatic cell lineages is also essential to incorporate best immunosuppressive strategy to facilitate their engraftment.

## Immunogenic Functionality of PSC-Derived Immune Cells

Although donor-specific iPSC lines and their cellular derivatives are not expected to be inherently immunogenic, iPSC-derived cellular derivatives are expected to be fully functional, which in the case of iPSC-derived immune cells is orchestration of all the immunological parameters associated with the immune cell lineage of choice. Donor-derived immune cells, such as DC and T cells have been used to develop DC-based cancer vaccines and tumor antigen-specific T cell receptor (TCR)/chimeric antigen receptor (CAR) engineered antitumor T cells that have produced remarkable clinical outcomes in cancer patients (52–58). However, since donor-derived mature T cells possess endogenous TCR and the DC derived from cancer patients might be compromised in their immunogenicity profile due to tumor-induced immune suppression mechanisms, availability of donor-specific naive immune effectors with customized functional profile could significantly improve the efficacy of current immunotherapy approaches (58, 59). In this context, iPSC-derived immunogenic APC would be very useful for developing personalized cancer immunotherapy approaches, not only as standalone cancer vaccines but also as an adjuvant for T cell-based adaptive immunotherapy approaches. Donor-specific tolerogenic APC on the other hand would be useful for the treatment of autoimmune diseases. Similarly, donor-specific naive antitumor T cells, generated with TCR- or CAR-based approaches, could improve the efficacy of adoptive transfer-based cancer immunotherapy approaches (58, 60, 61). This would be of special significance for the elderly where the quantity as well as the quality of immune effectors is compromised with age.

A significant progress has been made toward generating effector T cells, APC, NK cells, and other blood lineages from the hESC and iPSC lines (62–65). Functional APC-derived from ESC and iPSC lines have been shown to effectively acquire and process the antigens and present antigenic peptide epitopes to T cells through traditional antigen presentation pathways as well as through cross-presentation routes (62, 66, 67). Customized antitumor T cells have also been derived from hESC and iPSC lines *via* tumor antigen-specific TCR and CAR engineering approaches, and these T cells have been shown to exhibit antigen-specific effector function (68–70). Donor-specific NK cells have also been derived from hESC and iPSC lines and these cells have also been shown to exhibit effector function *in vitro* as well as *in vivo* (63, 71). While fibroblasts remain one of the preferred cells for derivation of donor-specific iPSC lines, as mentioned before, lineage specific models could be useful to systematically characterize the inherent immune-reactivity of hPSC lines and somatic cells derived from them, and also for characterizing the molecular and functional profile of iPSC-derived somatic cells. For example, as mentioned before, human DC-derived iPSC lines have provided useful insights toward the inherent immunogenicity profile of human iPSC lines (14) and the iPSC-APC derived from them could also be useful for systematically characterizing their molecular as well as functional profile, utilizing autologous human DC as controls.

## Conclusion

Since the isolation of first hESC lines and especially the derivation of donor-specific iPSC lines, regenerative medicine field has progressed at an exponential pace. Methods have been developed to generate donor-specific iPSC lines that are free of integrating viral vectors from different somatic cell lineages, including terminally differentiated primary immune cells, and different types of functional somatic cells have been derived, some of which have even advanced to the clinical trials stage. However, several challenges, such as the inherent immunogenicity of iPSC lines, have also been identified that need to be addressed. Since iPSC lines derived from different somatic cells and with different iPSC derivation methods can harbor different genetic and epigenetic signatures, it is necessary to fully characterize the mechanism of development and maintenance of pluripotency to generate iPSC lines with stable genomic architecture. This is important given that incomplete reprogramming could generate a neoantigen repertoire that could translate into enhanced immunogenicity of iPSC lines. Furthermore, while inherent immunogenicity of iPSC lines is not desired, iPSC-derived immune cells need to be fully functional. Therefore, according to the somatic cell lineage of choice, appropriate models need to be developed to characterize their molecular, cellular, as well as functional profiles. Addressing these issues is critical for developing safe and effective CRT.

## Author Contributions

AC conceived and wrote the manuscript.

## Conflict of Interest Statement

The author declares that the research was conducted in the absence of any commercial or financial relationships that could be construed as a potential conflict of interest. The reviewer, FR, and handling editor declared their shared affiliation, and the handling editor states that the process nevertheless met the standards of a fair and objective review.

## Abbreviations

hPSCs, human pluripotent stem cells; hESCs, human embryonic stem cells; EB, embryoid bodies.
